# Retroperitoneal Soft Tissue Xanthogranulomatous Pseudocyst: A Report of a Rare Case and Literature Review

**DOI:** 10.7759/cureus.97325

**Published:** 2025-11-20

**Authors:** Raj M Dongol, Mohadese Behtaj, Tapan M Bhavsar, Paul P Lin, Abiye Kassa

**Affiliations:** 1 Department of Pathology and Laboratory Medicine, George Washington University School of Medicine and Health Sciences, Washington, DC, USA; 2 Department of Surgery, George Washington University School of Medicine and Health Sciences, Washington, DC, USA

**Keywords:** foamy histiocyte, infiltration, pseudocyst, retroperitoneal xanthogranuloma, soft tissue

## Abstract

Xanthogranulomatous conditions are rare entities. Xanthogranulomatous inflammation, primarily involving the retroperitoneal soft tissue without visceral organ involvement, is very rare. Pseudocyst formation as a sequela of this condition is even rarer and has been barely reported in the literature. In the literature review, only a few cases of retroperitoneal xanthogranulomatous pancreatic (visceral) pseudocyst were reported, with no reports of retroperitoneal primary soft tissue origin.

We report a very rare case of a large, benign, primary soft tissue retroperitoneal xanthogranulomatous pseudocyst in a 33-year-old woman who presented with abdominal pain and significant weight loss. Complete excision of the mass was possible due to the lack of infiltration. Histopathologic examination showed xanthogranulomatous inflammation, consisting of sheets of cluster of differentiation 68 (CD68)-positive, foamy, lipid-laden histiocytes (xanthoma cells) surrounding areas of necrosis with pseudocyst formation. Other immunostains were negative, including CD1a, S100, and B-Raf proto-oncogene (BRAF) V600E, supporting the sporadic nature without multisystem involvement. Although the histology and immunostains for vascular markers (D2/40 and CD31) did not identify a vascular tumor, the possibility that this xanthogranulomatous inflammation originated from a ruptured and infected retroperitoneal lymphangioma cannot be entirely excluded. Retroperitoneal soft tissue origin, extensive adherence to multiple organs without their involvement, absence of adjacent tissue infiltration despite being large, and significant weight loss, despite being benign, are unique features in the present case. Retroperitoneal xanthogranuloma can become a critical condition, given the adequate space in the retroperitoneum, allowing it to grow until large enough to manifest clinically, and its potential for adjacent tissue infiltration even when small. In addition, the clinical presentation is often non-specific, and radiologic diagnosis is difficult. Considering all these factors, it is prudent to include it in the differential diagnosis, as early removal of the mass is the only way to prevent local tissue infiltration and halt the aggressive nature of this disease.

## Introduction

Xanthogranulomatous conditions are rare entities characterized by a collection of lipid-laden macrophages, known as foamy histiocytes. Causes include inflammation, infection, inherited lysosomal disorders, or histiocytic processes, including Langerhans and non-Langerhans cell histiocytosis [1]. The non-Langerhans cell histiocytoses associated with xanthogranulomatosis include Erdheim-Chester disease, Rosai-Dorfman disease, juvenile xanthogranuloma, and hemophagocytic lymphohistiocytosis [1]. Similarly, associated inherited lysosomal disorders include Niemann-Pick and Gaucher disease [1]. The kidneys and the gallbladder are common sites for xanthogranulomatous inflammation [2]. Xanthogranulomatous inflammatory conditions are pseudotumorous lesions that closely mimic malignancy clinically, radiologically, and pathologically. Xanthogranuloma is a localized benign lesion of histiocytic origin, whereas the term xanthogranulomatous disease is used to collectively describe xanthogranulomas that are localized, as well as those associated with multisystem involvement.

Xanthogranulomatous inflammation of the retroperitoneal primary soft tissue is rare [3]. It can occur as localized xanthogranulomatous solid, cystic, or pseudocystic masses, or as xanthogranulomatous disease with multisystem involvement, as in Erdheim-Chester disease. We report a very rare case of a large, benign, chronic, necrotizing, retroperitoneal soft tissue xanthogranulomatous pseudocyst in a 33-year-old female, who was treated with complete excision of the mass.

## Case presentation

**Figure 1:**
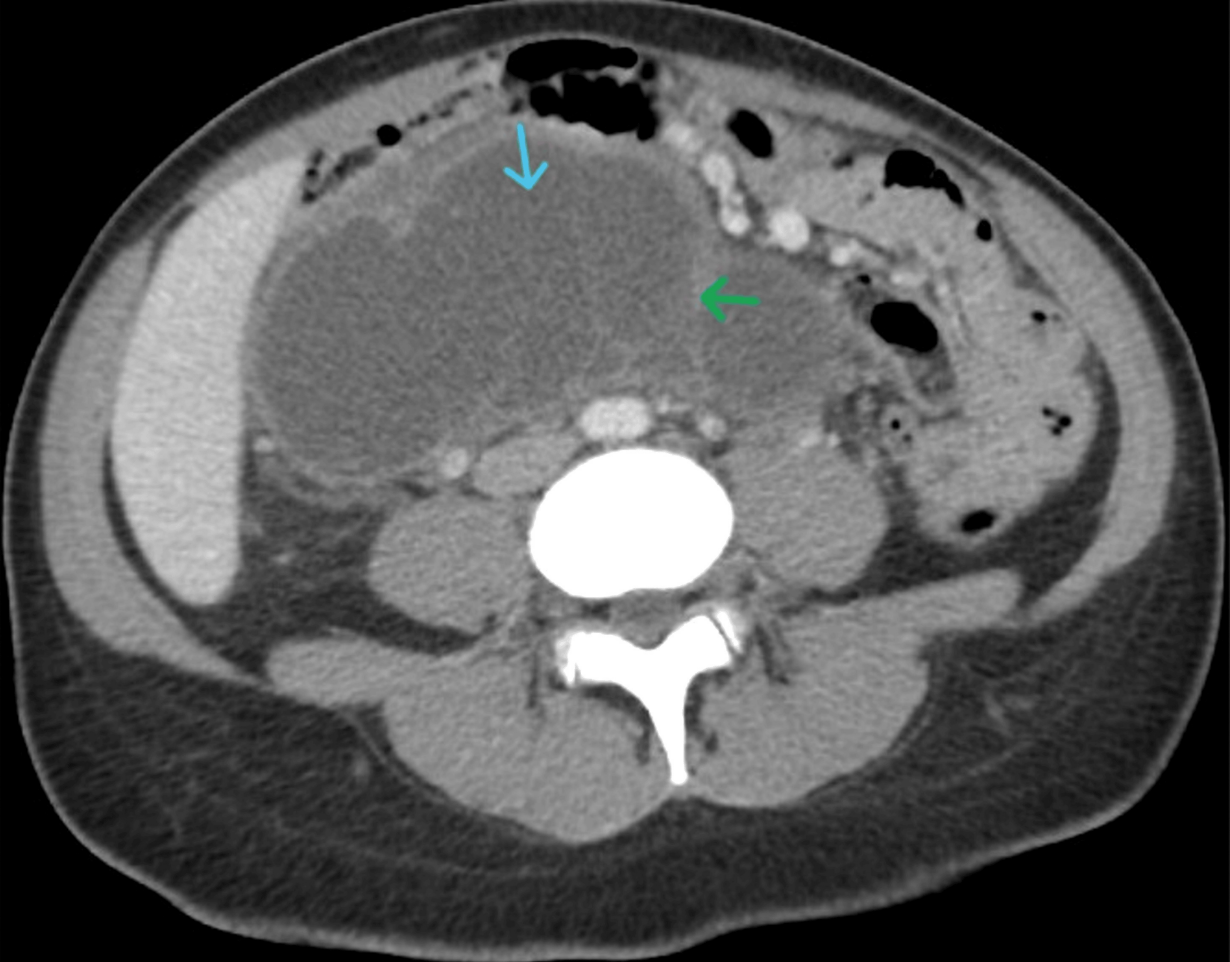
Contrast-enhanced CT of the abdomen and pelvis showing a large, complex, septate (green arrow), cystic (blue arrow) retroperitoneal mass The retroperitoneal mass is located in the mid-abdomen, extending from the right upper quadrant to just below the iliac crest, displacing the pancreas and duodenum anteriorly, and without a significant solid component. Mild enhancement is noted along the walls and septations.

This is the case of a 33-year-old female who presented to the Emergency Department with nausea and loss of appetite for three weeks, along with a gradual onset of mild to moderate central abdominal pain radiating to the back for two weeks. The pain had worsened over the last 24 hours and was associated with subjective fever and chills. She had a history of unintentional, significant weight loss (10 lbs over three weeks). She was hemodynamically stable. Systemic examination was unremarkable, except for mild abdominal distension and tenderness over the mid to right side of the abdomen. Laboratory investigations were unremarkable, with a normal carcinoembryonic antigen (CEA) level of <2 ng/mL. Computed tomography (CT) of the abdomen and pelvis showed a large (15.8 × 13.2 × 6.6 cm), complex, septate cystic mass in the mid-abdomen, extending from the right upper quadrant to just below the level of the iliac crest (Figure 1). The mass displaced the pancreas and duodenum anteriorly. There was no significant solid component of the mass. The walls and septations showed mild enhancement. Radiological differential diagnoses included lymphangioma, mesenteric cyst, and pancreatic pseudocyst. This was further evaluated by upper endoscopic ultrasonography (EUS), which showed a retroperitoneal, septate cyst measuring 16 × 9.9 cm in the periduodenal space, with moderate extrinsic deformity of the first and second parts of the duodenum. Diagnostic aspiration yielded 60 mL of opaque, brown, thin fluid, which was sent for cytology and microbiology. Cytology did not show any atypical or malignant cells. Culture grew *Streptococcus*. Fluid triglyceride level was 4,089 mg/dL. Upper gastrointestinal barium study was within normal limits.

The patient underwent laparoscopic retroperitoneal mass excision, which was converted to open surgery due to the large size of the mass and its adherence to major vessels. No suspicious liver lesions or evidence of metastatic disease were identified. A large, central, retroperitoneal, complex cystic/solid mass, measuring 18 cm at its greatest dimension, was found, extending from the right to the left retroperitoneum. The mass compressed and displaced the duodenum, pancreas, and stomach anteriorly and to the left. Inferiorly, it displaced the transverse colon and mesocolon. The lesion was firmly adherent to the duodenum and base of the mesentery, as well as to the transverse colon, mesocolon, superior mesenteric vessels, portal vein, inferior vena cava, renal vessels, aorta, right kidney, gallbladder, and porta hepatis. On entering the mass, copious turbid chylous fluid, without bilious content, was evacuated, allowing complete removal of the lesion. The intraoperative and postoperative courses were uneventful.

Grossly, the resected mass consisted of an unoriented, disrupted cystic lesion measuring 11 × 9.4 × 3 cm, surrounded by a pink-red capsule with diffuse erythematous adhesions and a scant amount of attached adipose tissue. Opening of the cystic lesion revealed multicystic spaces filled with a minimal amount of clear, pink-red serous fluid and a large amount of yellowish, friable, soft material. The cyst wall lining was pink-tan to yellow and diffusely trabecular, with no firm nodules. Sectioning revealed a pink-tan, soft cut surface with diffuse areas of erythema and yellowish deposits.

**Figure 2:**
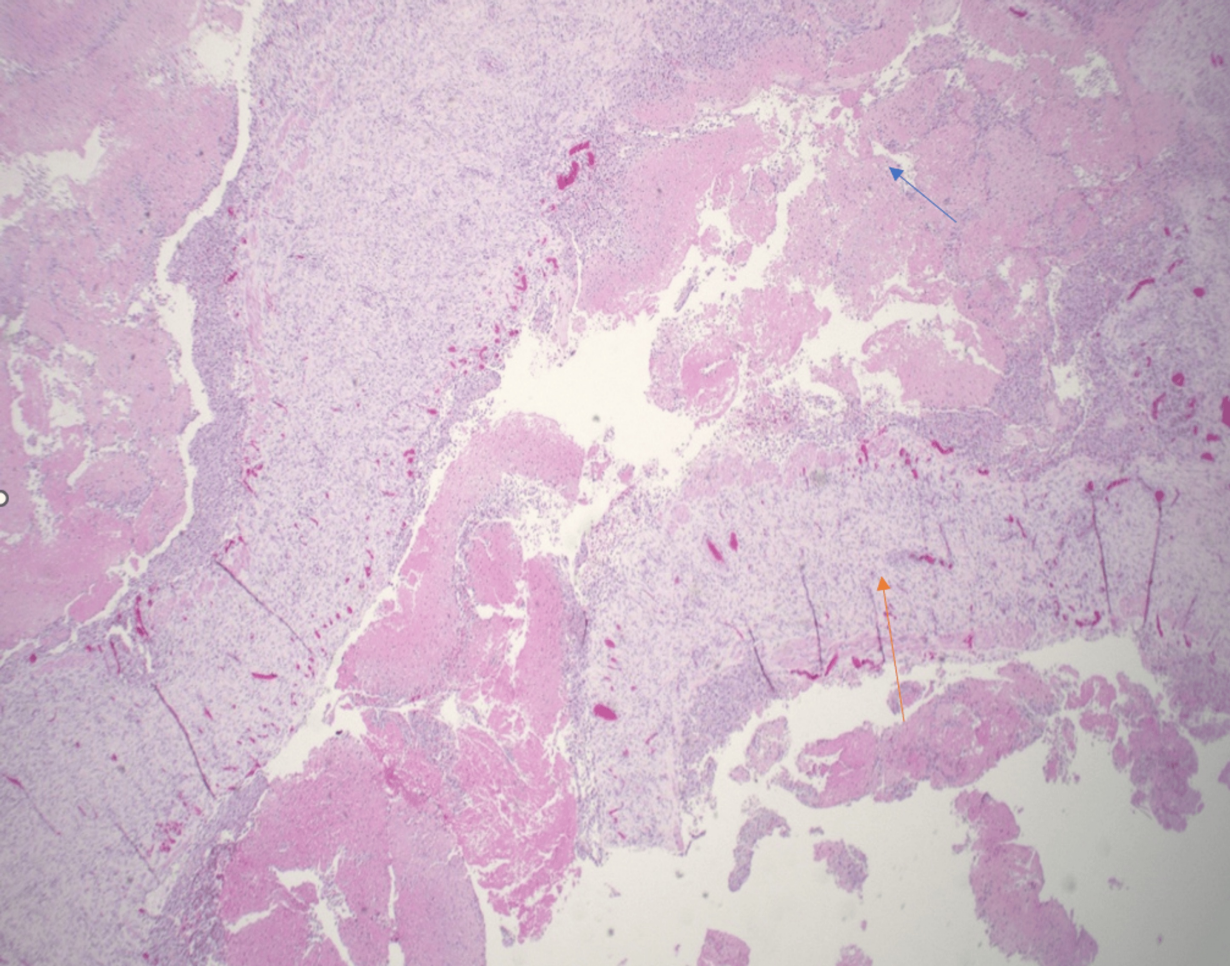
Multicystic structure with central necrosis (blue arrow) and fibrous septae (orange arrow) (H&E stain, 20×)

**Figure 3:**
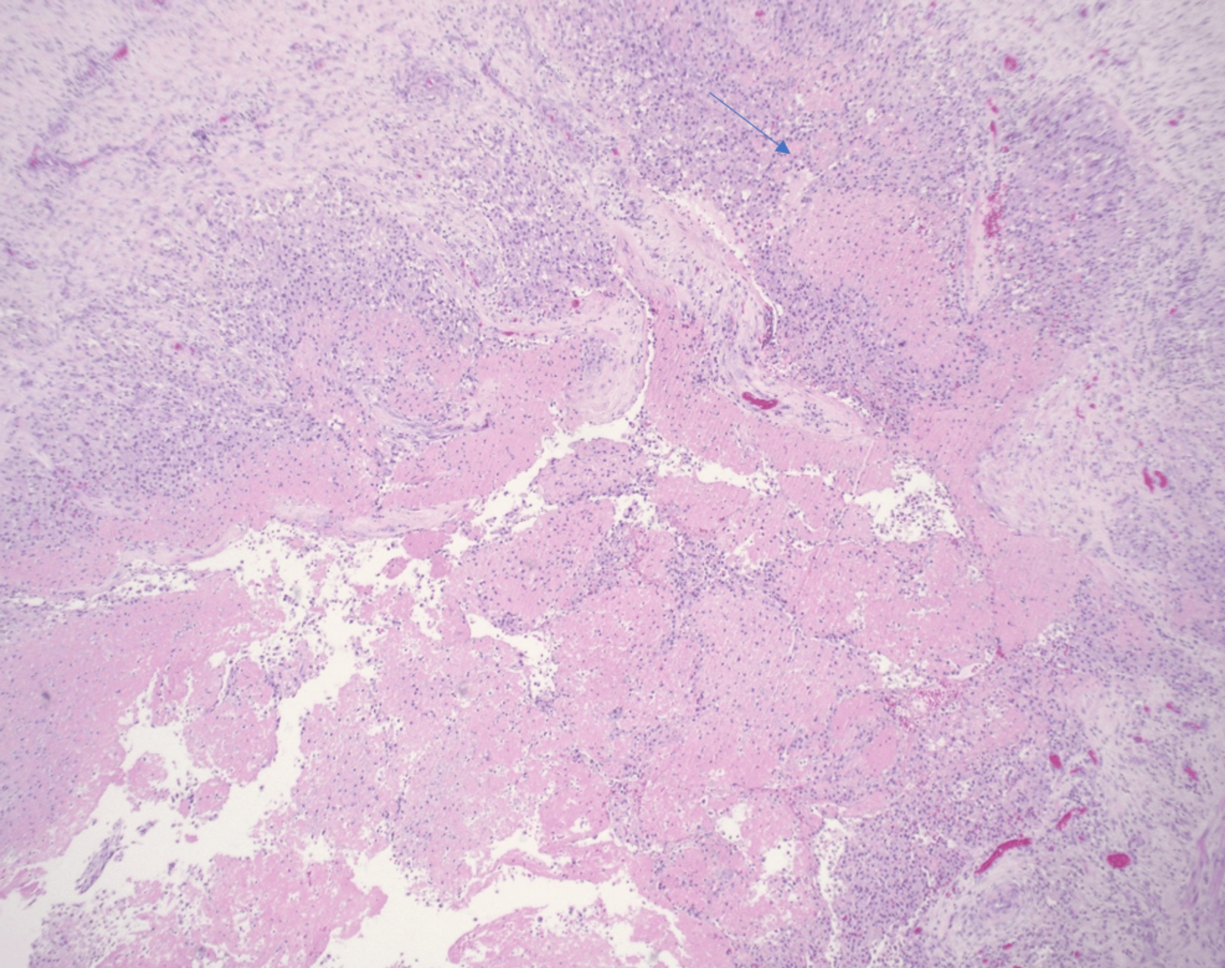
Xanthogranulomatous inflammation (arrow) lining the fibrous septae with central necrosis (H&E stain, 40×)

**Figure 4:**
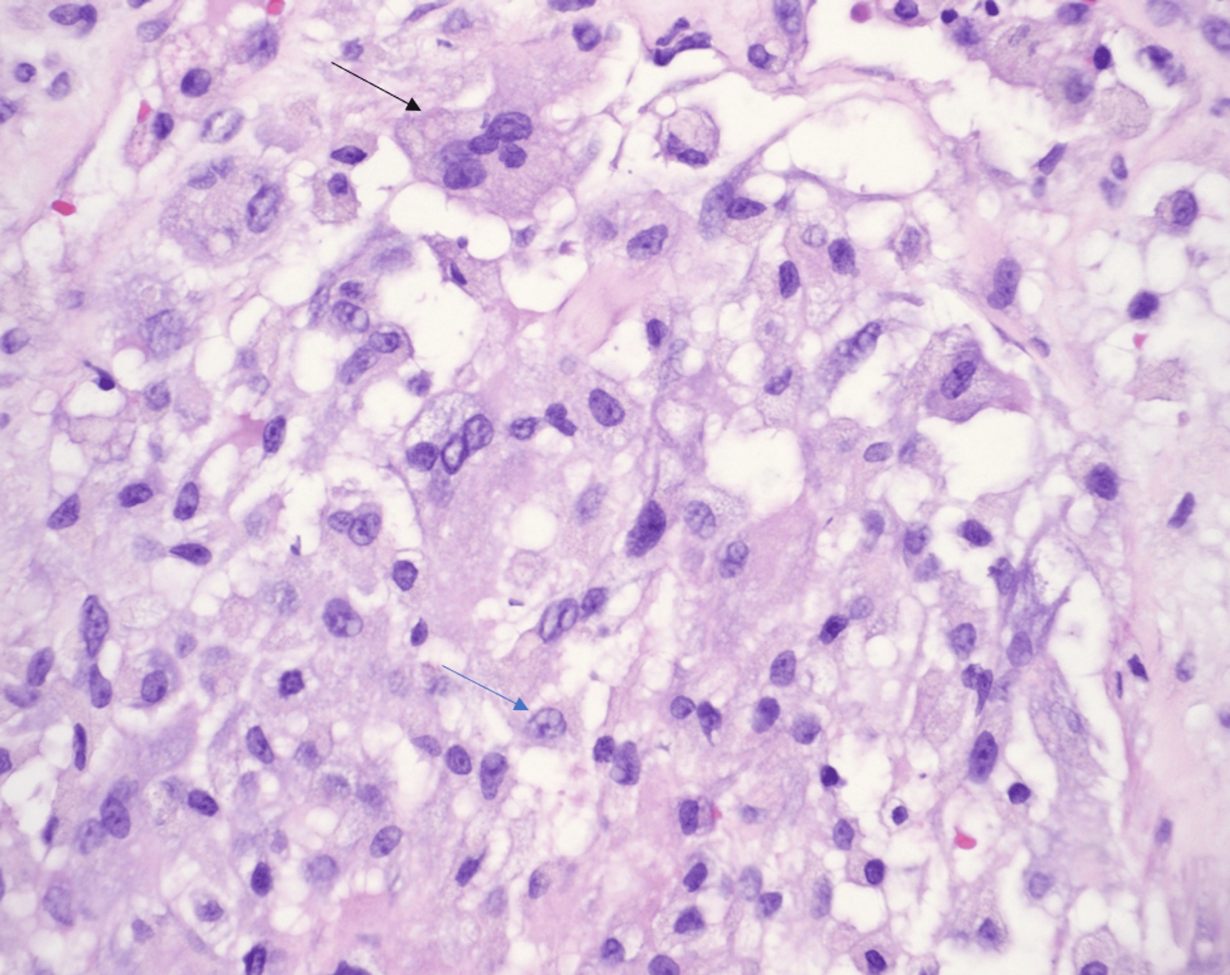
High power magnification showing lipid-laden foamy histiocytes (blue arrow) and giant cells (black arrow) lining the pseudocytic structure (H&E stain, 400×)

**Figure 5:**
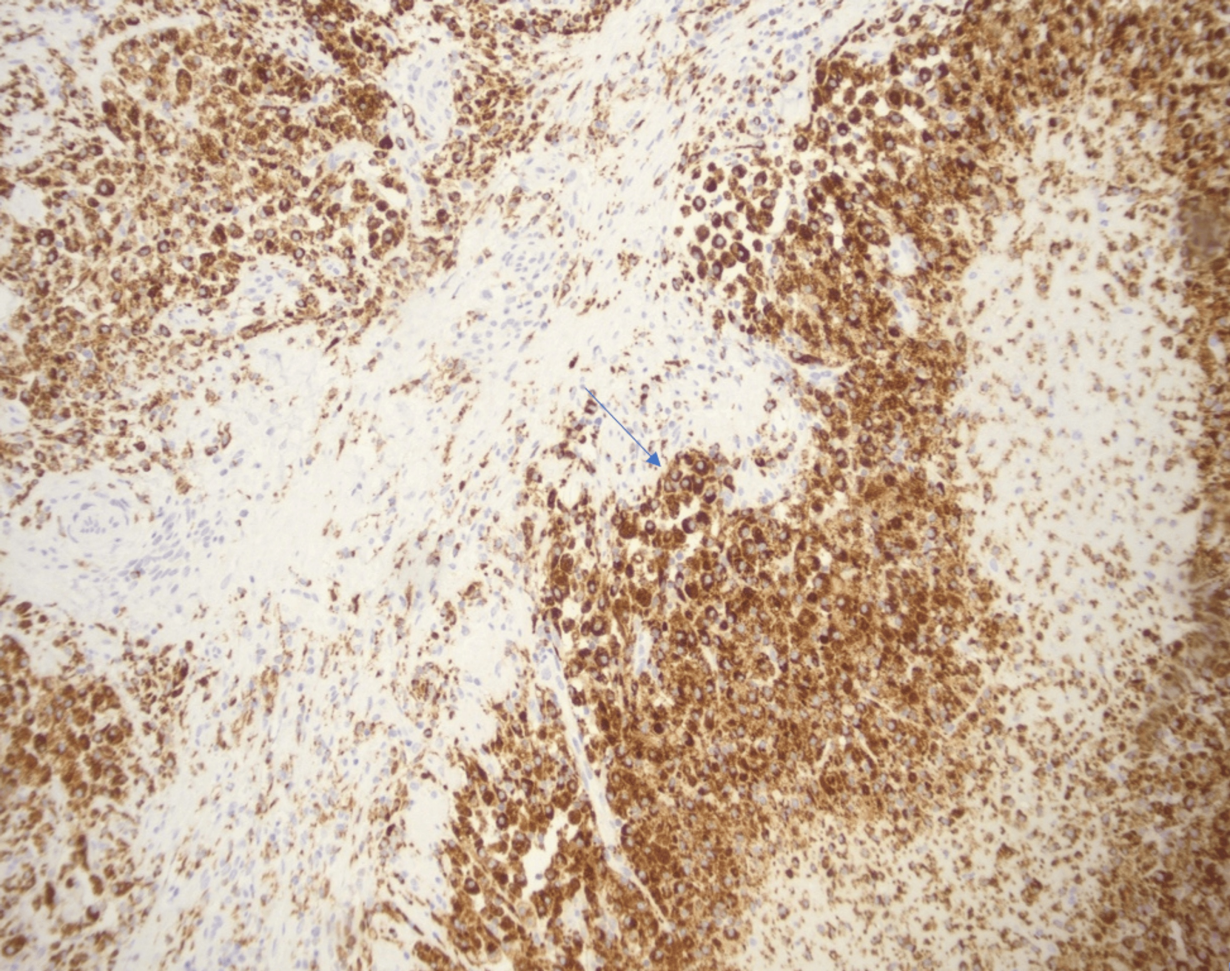
CD68 immunohistochemical stain highlights sheets of xanthoma cells (arrow) CD: cluster of differentiation

**Figure 6:**
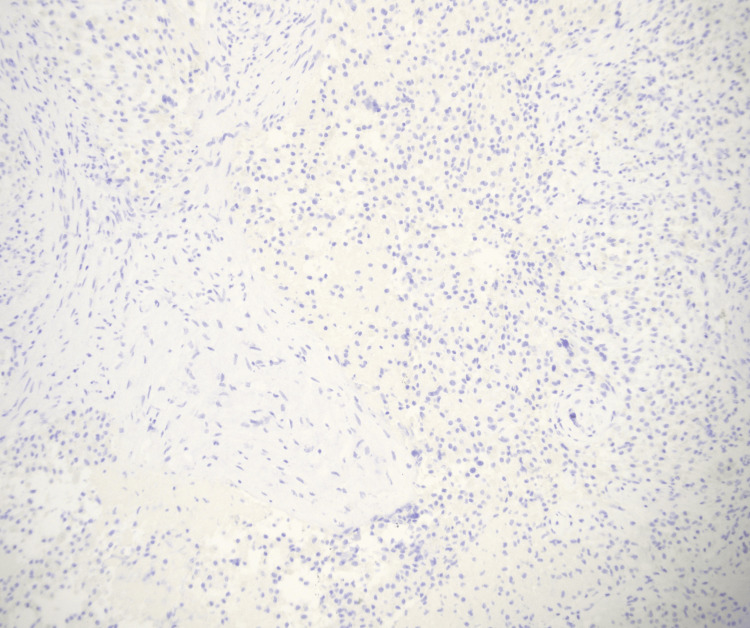
Histiocytes are CD1a negative ruling out Langerhans cell histiocytosis CD: cluster of differentiation

Histologically, it showed multiple foci of central necrosis surrounded by clusters of foamy histiocytes, granulation tissue, and chronic inflammation (Figures 2-4). Immunostains for cluster of differentiation 68 (CD68) (Figure 5) and CD45 highlighted the histiocytes. Immunostain for desmin showed the smooth muscle bundles. The cyst did not show any clear endothelial lining with CD31, CD34, and D2-40 immunostains. It was negative for CD1a, signifying the absence of Langerhans cell histiocytes (Figure 6).

**Figure 7:**
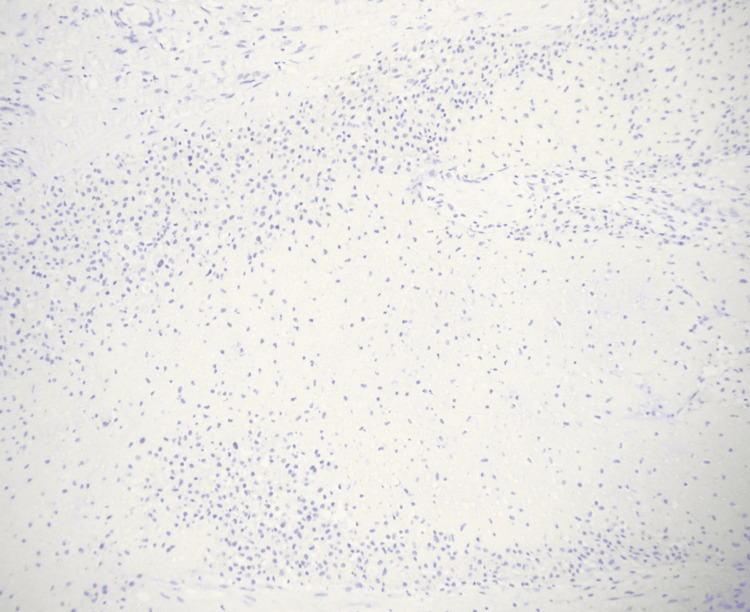
Histiocytes are negative for S100 immunohistochemical stain ruling out Rosai-Dorfman disease

**Figure 8:**
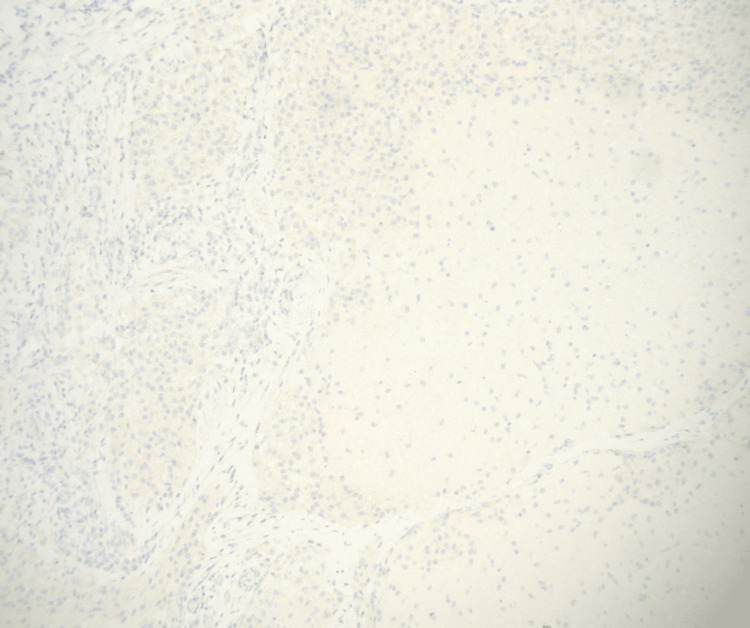
Histiocytes are negative for BRAF V600E immunohistochemical stain making Erdheim-Chester disease less likely BRAF: B-Raf proto-oncogene; V600E: valine (V) amino acid at position 600 replaced by glutamic acid (E)

Immunostains for AE1/AE3 (cytokeratin AE1/AE3), epithelial membrane antigen (EMA), Wilms tumor 1 (WT1), paired box gene 8 (PAX8), inhibin, alpha-fetoprotein (AFP), octamer-binding transcription factor 4 (OCT4), calretinin, CD117, Melan-A, human melanoma black 45 (HMB45), B-Raf proto-oncogene (BRAF) V600E (Figure 7), S100 (Figure 8), and CD30 were negative, excluding the possible origin of any known malignant tumor cells. Immunostain for p53 showed wild-type staining. In situ hybridization for Epstein-Barr virus (EBV) was negative. Gomori methenamine silver (GMS) and acid-fast bacilli (AFB) stains were negative for fungal organisms and acid-fast bacilli, respectively.

Therefore, the lesion was negative for known malignancy, autoimmune disease, fungal infection, or tubercle bacilli infection. The morphology and immunoprofile were those of a benign chronic inflammatory process, consistent with chronic necrotizing xanthogranulomatous inflammation forming a pseudocystic structure due to the central necrosis.

## Discussion

Retroperitoneal xanthogranuloma is a rare primary soft tissue tumor, most of which are benign [3]. Retroperitoneal xanthogranulomatous pseudocyst is an extremely rare finding, with only a few cases of retroperitoneal xanthogranulomatous pancreatic pseudocysts reported in the literature [4]. Oberling first reported retroperitoneal xanthogranuloma in 1935 [5]. Extrapancreatic xanthogranulomatous cysts or pseudocysts are exceedingly rare and have been scarcely reported. We present a rare case of a large retroperitoneal xanthogranulomatous pseudocystic mass, most likely arising from chronic retroperitoneal soft tissue infection, as aspirated fluid from the cystic mass grew *Streptococcus* on culture, leading to retroperitoneal xanthogranuloma. Necrosis within this xanthogranuloma may have contributed to the formation of a multiseptated pseudocystic structure. Given the high triglyceride level in the cyst content, an alternative origin for this pseudocyst could be a ruptured and infected cystic lymphangioma, with secondary xanthogranulomatous inflammation, necrosis, and complete obliteration of the endothelial lining.

The basic pathogenesis of xanthogranulomatous inflammation involves recurrent inflammation or infection, leading to chronicity, which is grossly identifiable as granulation tissue and necrotic debris [1]. During this process, inflammatory cells, including histiocytes, accumulate, accompanied by neovascularization and hemorrhage [1]. This may result in abnormal lipid transport, lymphatic obstruction, and immune dysfunction [2]. Histiocytes engulf erythrocytes and lipids, forming lipid-laden foamy histiocytes, also known as xanthoma cells [1]. Grossly, these appear as golden or bright yellow nodules [2]. In cases with extensive necrotic and cystic degeneration, as in the present case, pseudocyst formation can occur, with yellowish deposits present within the wall, septa, or debris. These foamy histiocytes exhibit a wide range of immunohistochemical staining, based on their origin. All histiocytes are CD68-positive [1]. Non-Langerhans cell histiocytes are negative for CD1a and Langerin (CD207) and lack Birbeck granules, unlike Langerhans cell histiocytes [6]. Histiocytes in Erdheim-Chester disease are S100-negative, unlike histiocytes in Rosai-Dorfman disease and Langerhans cell histiocytosis [1,6]. Erdheim-Chester disease is a multisystem disorder, commonly affecting the long bones and, less frequently, the heart, lungs, blood vessels, skin, and retroperitoneum [1]. Rosai-Dorfman disease presents with massive lymphadenopathy, with or without extranodal involvement, and is characterized histologically by emperipolesis - the phagocytosis of intact adjacent cells, including lymphocytes, by histiocytes [6].

Xanthogranulomatous processes can infiltrate adjacent organs, resulting in tissue destruction by foamy histiocytes, and leading to granuloma formation and collagen deposition [1]. The present lesion can be distinguished from true malignant pathologies based on its unique features. It differs from inflammatory malignant fibrous histiocytoma in terms of its inconspicuous vascularity, moderate collagen content, absence of neutrophils, and lack of nuclear atypia [7]. Similarly, it is distinguished from inflammatory fibrosarcoma by its relatively low plasma cell population, the presence of abundant foamy histiocytes, and the absence of nuclear atypia [7]. Briffod et al. described a case of true malignant fibrous histiocytoma of the sacrum, presenting as a retroperitoneal xanthogranulomatous cystic mass with osteolytic lesions in 1982; it demonstrated histological evidence of cellular atypia, along with aggregates of xanthoma cells and inflammatory cells [8]. In the present case, there was no evidence of neutrophils, plasma cells, or nuclear atypia. We concluded that the lesion represents a non-visceral soft tissue retroperitoneal xanthogranuloma, resulting in a xanthogranulomatous pseudocyst, based on a diagnosis of exclusion, and suggest that this entity should be included in the differential diagnosis of large retroperitoneal masses without a clear radiological origin. Large masses should be sampled generously to identify the tissue of origin if possible, and to rule out malignancy, which is critical for determining prognosis.

Large and extensively necrotic xanthogranulomatous pseudocystic masses, as seen in this case, are unusual. Despite its size, the mass did not infiltrate adjacent structures, allowing complete excision without damage to surrounding organs. It was compressing and adherent to the duodenum, pancreas, stomach, and vessels. The compression of surrounding structures led to symptoms such as abdominal pain, nausea, anorexia, and weight loss. Despite being benign, the mass caused significant weight loss, mimicking malignancy. The patient’s weight loss was likely secondary to duodenal compression and partial gastric outlet obstruction. In contrast, small xanthogranulomas often remain undiagnosed due to the absence of compressive symptoms; however, tumors near the urinary tract, or adherent to intestinal loops, are usually detected early due to urinary symptoms or intestinal obstruction [2,9]. In the present case, the tumor was sufficiently large to cause clinical symptoms. Diagnostic tools remain limited, and xanthogranuloma is challenging to detect due to the lack of specific clinical manifestations.

A review of the PubMed database identified 64 published articles on retroperitoneal xanthogranuloma and related conditions. Among them, 66 cases of retroperitoneal xanthogranuloma, both benign and malignant, were reported. No information was available for 26 cases. Of the remaining 40 cases, 13 were malignant and 27 were benign. Among the benign cases, six were associated with Erdheim-Chester disease, and two with juvenile xanthogranulomatosis. The remaining 19 benign cases were sporadic and isolated: five infiltrated the ureter, five involved the kidney (with one also associated with visceral eosinophilic granuloma, and another with expanding renal subcapsular hematoma), one was a mesenteric retroperitoneal xanthogranuloma infiltrating the duodenum, one had recurrence, and one underwent malignant transformation [9-18]. These cases most commonly present with abdominal pain, a palpable mass, or urinary symptoms. No cases of retroperitoneal xanthogranulomatous pseudocyst formation were identified, except for pancreatic xanthogranulomatous pseudocysts. Therefore, the present case represents the first sporadic, large retroperitoneal xanthogranulomatous pseudocyst arising from non-visceral retroperitoneal soft tissue. Selected cases of sporadic benign retroperitoneal xanthogranulomatous lesions are summarized in Table 1.

**Table 1:** Reported sporadic benign retroperitoneal xanthogranulomatous conditions

Author (year)	Country	Age/Sex	Size (cm)	Presenting symptom	Diagnosis	Treatment
Waller et al. (1957) [10]	United States	51/F	8.5	Right flank mass	Retroperitoneal xanthogranuloma involving right kidney, associated with visceral eosinophilic granuloma	Excision of mass with right nephrectomy
Pear (1970) [11]	United States	73/M	15	Unknown	Retroperitoneal xanthogranuloma in the mesentery infiltrating the wall of the 4th part of the duodenum with adenocarcinoma of the stomach	Excision of mass with duodenum, part of jejunum, and stomach
Gup (1972) [12]	United States	56/F	7 × 7	Right lower abdominal pain	Retroperitoneal xanthogranuloma infiltrating the ureter with endometriosis	Excision of mass with part of the ureter; intraperitoneal ureteral transplantation
Cozzutto et al. (1978) [18]	Italy	9/M	Unknown	Abdominal lump, fever, anorexia, weight loss	Retroperitoneal xanthogranuloma which recurred and later transformed to malignant fibroxanthosarcoma	Excision of the tumor
Hennessy et al. (1990) [9]	Australia	73/F	4	Left loin pain	Retroperitoneal xanthogranuloma involving the upper left ureter	Excision of mass, kidney, and upper ureter
Suzuki et al. (1992) [13]	Japan	51/M	2	Right flank pain	Retroperitoneal xanthogranuloma adhering to the right ureter	Excision of mass with partial ureter excision and ureteroureterostomy
Kondo et al. (1998) [14]	Japan	57/M	5	Left flank pain	Retroperitoneal xanthogranuloma with extrinsic stenosis of the left ureter	Exploration and biopsy; the mass decreased in size and patient's condition improved
Salehin et al. (2010) [15]	Germany	24/F	Unknown	Unknown	Retroperitoneal xanthogranuloma	Excision of mass; recurrence after 2 years
Kubota et al. (2015) [16]	Japan	23/M	5	Abdominal pain	Retroperitoneal xanthogranulomatous pancreatic pseudocyst	Distal pancreatectomy, partial colectomy, total gastrectomy with splenectomy
Dongol et al. (2025) (present case)	United States	32/F	18	Abdominal pain, loss of appetite, significant weight loss	Primary soft tissue retroperitoneal xanthogranulomatous pseudocyst	Excision of mass

Treatment varies based on individual cases. Surgical excision is generally the preferred approach, as it prevents local spread of the xanthogranuloma [2]. Adjuvant radiotherapy or chemotherapy may be indicated, depending on the specific diagnosis [19]. Some cases respond to steroid therapy [20], and spontaneous resolution has also been reported [2]. In the present case, complete tumor excision was achieved, resulting in cure. Relapse may occur, even after treatment, with or without malignant transformation; therefore, close follow-up is recommended.

## Conclusions

Primary soft tissue retroperitoneal xanthogranulomatous conditions are rare. We report an exceptionally rare case of a large, benign, primary soft tissue retroperitoneal xanthogranulomatous pseudocyst in a 33-year-old female, without adjacent tissue infiltration, which was successfully treated with complete surgical excision. Retroperitoneal masses may remain asymptomatic for long periods due to the ample space in the retroperitoneum, until they grow large enough to cause clinical symptoms. Xanthogranuloma is a pathological diagnosis, and, by nature, it has the potential for aggressive infiltration of adjacent tissues. Prompt surgical exploration and excision of the mass is, therefore, crucial to prevent local tissue involvement.
